# Characterisation and natural progression of SARS-CoV-2 infection in ferrets

**DOI:** 10.1038/s41598-022-08431-6

**Published:** 2022-04-05

**Authors:** Gough G. Au, Glenn A. Marsh, Alexander J. McAuley, Suzanne Lowther, Lee Trinidad, Sarah Edwards, Shawn Todd, Jennifer Barr, Matthew P. Bruce, Timothy B. Poole, Sheree Brown, Rachel Layton, Sarah Riddell, Brenton Rowe, Elisha Soldani, Willy W. Suen, Jemma Bergfeld, John Bingham, Jean Payne, Peter A. Durr, Trevor W. Drew, Seshadri S. Vasan

**Affiliations:** Commonwealth Scientific and Industrial Research Organisation, Australian Centre for Disease Preparedness, 5 Portarlington Road, Geelong, VIC 3220 Australia

**Keywords:** Viral infection, SARS-CoV-2, Experimental models of disease

## Abstract

Severe acute respiratory syndrome coronavirus 2 (SARS-CoV-2) is responsible for the infectious disease COVID-19, which has rapidly become an international pandemic with significant impact on healthcare systems and the global economy. To assist antiviral therapy and vaccine development efforts, we performed a natural history/time course study of SARS-CoV-2 infection in ferrets to characterise and assess the suitability of this animal model. Ten ferrets of each sex were challenged intranasally with 4.64 × 10^4^ TCID_50_ of SARS-CoV-2 isolate Australia/VIC01/2020 and monitored for clinical disease signs, viral shedding, and tissues collected post-mortem for histopathological and virological assessment at set intervals. We found that SARS-CoV-2 replicated in the upper respiratory tract of ferrets with consistent viral shedding in nasal wash samples and oral swab samples up until day 9. Infectious SARS-CoV-2 was recovered from nasal washes, oral swabs, nasal turbinates, pharynx, and olfactory bulb samples within 3–7 days post-challenge; however, only viral RNA was detected by qRT-PCR in samples collected from the trachea, lung, and parts of the gastrointestinal tract. Viral antigen was seen exclusively in nasal epithelium and associated sloughed cells and draining lymph nodes upon immunohistochemical staining. Due to the absence of clinical signs after viral challenge, our ferret model is appropriate for studying asymptomatic SARS-CoV-2 infections and most suitable for use in vaccine efficacy studies.

## Introduction

By the 6th of January 2022, more than 297.6 million cases of coronavirus disease 2019 (COVID-19) have been confirmed worldwide, responsible for greater than 5.46 million deaths globally^1^. The outbreak was first reported in Wuhan, China in December 2019, where the causative agent was quickly identified as a new coronavirus (2019-nCoV) likely to have originated from bats^2^. The virus was subsequently renamed to “severe acute respiratory syndrome coronavirus 2”, SARS-CoV-2^3^ due to the close genetic similarity of this virus to the highly pathogenic coronavirus responsible for the SARS outbreak of 2002^4^.

Coronaviruses are enveloped, positive-sense, single-stranded RNA viruses belonging to the family *Coro-naviridae* of the *Nidovirales* order^5,6^. Divided into four genera, *Alphacoronavirus*, *Betacoronavirus*, *Gam-macoronavirus,* and *Deltacoronavirus*, the alpha- and betacoronaviruses are often associated with mild respiratory illness in humans and gastroenteritis in animals^7^. Unlike the alpha- and betacoronaviruses, which have bat origins, gamma- and deltacoronaviruses are avian and pig associated^8^. The outbreak of SARS in 2002 in Guangdong province, China^9^ and then Middle East respiratory syndrome coronavirus (MERS-CoV) in Saudi Arabia in 2012^10^ highlighted the potential for coronaviruses to become highly pathogenic as they spillover from animal reservoirs into humans.

SARS-CoV-2, like SARS coronavirus (SARS-CoV), is transmissible via respiratory droplets and/or aero-sols as well as via direct contact, utilising angiotensin-converting enzyme 2 (ACE2) as a functional entry receptor to infect host cells^2,11^. Based on single-cell RNA expression profiling in normal human lungs, ACE2 is expressed in 0.64% of all human lung cells, with the majority of ACE2-expressing cells (83%) identified as type II alveolar cells^12^. ACE2 expressing cells also include type I alveolar epithelial cells, fibroblasts, endothelial cells, and macrophages, however, their ACE2-expressing cell ratios are relatively low and variable among individuals. Surface expression of ACE2 protein has previously been established in lung alveolar epithelial cells and enterocytes of the small intestine, as well as endothelial cells and arterial smooth muscle cells^13^. Epithelial cells of the oral mucosa and particularly the tongue also express high levels of ACE2 receptor, providing a potential route of virus entry^14^.

The clinical presentation of COVID-19 patients can range from asymptomatic to mild, to severe and critical^15,16^. Common symptoms at disease onset include fever, fatigue, dry cough, myalgia, and dyspnea^17^, while diarrhea, hemoptysis, headache, and sore throat are rare. Neurological manifestations in patients with COVID-19 not only involved the central nervous system but the peripheral nervous system as well, with impairment of taste and smell noted in some patients^18^. Mild disease varies from no symptoms to mild pneumonia, with severe disease characterised by moderate to severe pneumonia. Of 72,314 COVID-19 cases in mainland China, 81% were classified as mild, 14% severe, and 5% as critical, where the patient was in a state of respiratory failure, septic shock, and/or multiorgan failure^19^.

Reviewed by Cleary et al.^20^ and Muñoz-Fontela et al.^21^, there are a range of animal species sensitive to SARS-CoV-2 infection, including macaques, cats, ferrets, hamsters, and transgenic mice expressing human ACE2. Many of these models, however, only partially recapitulate symptoms of human COVID-19 cases, and disease in animals is generally mild^20,22^. Rhesus macaques have provided the most reliable models of COVID-19 so far with clinical, radiological, and histological evidence of acute respiratory distress syndrome^23,24^, as well as immunological evidence of animals being protected when rechallenged after primary SARS-CoV-2 exposure^25^. The main issues with non-human primate research is the lower throughput and additional ethical considerations associated with their advanced cognition.

Since ferrets were first discovered to be sensitive to influenza virus in the 1930s^26^, ferrets have been a mainstay of respiratory virus research, as they exhibit a cough reflex and also sneeze. They have proven to be useful virus transmission models for SARS-CoV-2, with independent researchers demonstrating spread of virus through the air between ferrets and via direct contact^27,28^. Most studies to date have focused on examining viral excretion in nasal washes, oral and rectal swab samples with few performing virological assessments of SARS-CoV-2 in a comprehensive panel of tissues post-infection. Additionally, it remains unclear whether there are any gender-based differences in disease severity among ferrets, as observed in humans^29^.

Our group together with many others have confirmed the susceptibility of ferrets to SARS-CoV-2 infection^27,28,30–34^. To further address the natural progression of the disease in ferrets and to trace the dissemination of SARS-CoV-2 in different organs and tissues, we conducted a serial sacrifice/time course study over 14 days following intranasal challenge of both male and female ferrets with 4.64 × 10^4^ TCID_50_ of SARS-CoV-2 isolate Australia/VIC01.

## Materials and methods

### Animal ethics statement

The animal work for this study was performed in the biosafety level-4 (BSL-4) large animal facility at the Australian Centre for Disease Preparedness (ADCP) in Geelong, Australia. All work with live animals was approved by the Commonwealth Scientific and Industrial Research Organisation (CSIRO) ACDP Animal Ethics Committee (AEC 1990) in accordance with guidelines from the Australian Code for the Care and Use of Animals for Scientific Purposes (8th Edition) and compliant with the Victoria State Prevention of Cruelty to Animals Act 1986 and Part 5 of the Prevention of Cruelty to Animals Regulations 2019. The facility has assurance from the US Office of Laboratory Animal Welfare Assurance (Legacy Assurance ID A5399-01). The study was carried out in compliance with the ARRIVE guidelines.

All animals were acclimatised for at least 7 days prior to entering the study, given food and water ad libitum, and monitored daily. Environmental enrichment was also provided in the cages during the study.

### Virus preparation and cells

SARS-CoV-2 (BetaCoV/Australia/VIC01/2020) was generously provided by the Victorian Infectious Diseases Reference Laboratory (VIDRL), The Peter Doherty Institute, Melbourne, Australia^35^. This isolate (passage 1 [P1]) was obtained from a nasopharyngeal swab of a 58-year-old male patient who presented with clinical signs, was PCR positive for SARS-CoV-2, and had a relevant travel history to Wuhan, China. The P1 isolate was passaged a single time on Vero E6 cells (Cat# NR-596, BEI Resources) to generate a working stock grown in DMEM (Cat# 11965092, Gibco, Thermo Fisher Scientific) with 10 mM HEPES (Gibco, Thermo Fisher Scientific), 2% (v/v) FBS (CellSera Australia, NSW) and 1% (v/v) antibiotic–antimycotic (Cat#15240112, Gibco, Thermo Fisher). The inoculated flasks were incubated at 37 °C with 5% CO_2_ and harvested once > 80% CPE was observed and clarified by centrifugation before pooling and storing at − 80 °C. Next-generation sequencing was performed as previously described^36^, and revealed that the working stock of virus was > 99.9749% in similarity with the originally published sequence (GISAID: EPI ISL 406844, GenBank Accession: MT007544.1). The virus was tested for the absence of mycoplasma contamination using the MycoSensor qPCR assay kit (Cat# 302106, Agilent) and virus titer determined by TCID_50_ assay on Vero E6 cells.

### Animals and study design

Ten male and ten female outbred ferrets (*Mustela putorius furo*) approximately four months of age were randomised into five groups (for humane killing on day 3, 5, 7, 9 or 14 post-infection). Standard husbandry of ferrets included the single administration of a commercially available canine vaccine Protech® C3 to prevent canine distemper at least 1 month prior to recruitment for study. Each ferret was implanted with a LifeChip Bio-Thermo (Destron Fearing, Eagan, MN, USA) microchip subcutaneously over the dorsal aspect of the scapula region. After a week of acclimatisation, and four days prior to virus challenge, ferrets were anaesthetised and baseline blood samples were collected for hematology (EDTA whole blood) and biochemistry (lithium heparin plasma), as well as nasal washes, oral swabs and rectal swabs collected. All ferrets were challenged intranasally (day 0) with SARS-CoV-2 VIC01 with a target dose of approximately 9 × 10^4^ TCID_50_ (back-titered to 4.64 × 10^4^ TCID_50_). The virus was administered dropwise alternating between each nare with a total volume of 0.5 mL of inoculum. After virus challenge, animals were monitored at least once daily (twice a day between days 3 and 10 post-infection or 48 h after last clinical observation if outside this range) for signs that included changes in body/microchip temperature, respiration, physical activity, lethargy, nasal discharge, sneezing, coughing, diarrhea or decrease in appetite. Animals that reached a predetermined humane endpoint for disease were immediately euthanased as described, irrespective of the study design schedule. Ferrets that remained on study were anaesthetised at indicated time-points for collection of clinical samples which included nasal washes, oral swabs, and rectal swabs. An additional rectal temperature was also collected while animals were anaesthetised. Based on the initial randomisation, at days 3, 5, 7, 9 and 14 days post virus challenge, two male and two female ferrets were humanely killed and a panel of tissues collected for histology and virological analysis.

### Sample collection and necropsy procedures

Ferrets were anaesthetised with an intramuscular injection of medetomidine and ketamine (0.05 mg/kg and 5 mg/kg, respectively) for inoculum administration and sampling procedures. For anaesthetic reversal, intramuscular injection of atipamezole (0.25 mg/kg) was used. At the terminal sampling point, ferrets were anaesthetised as described above and exsanguinated via cardiac puncture before humanely killed via injection of sodium pentobarbitone (up to 150 mg/kg) while under anaesthesia. After confirmation of death, a necropsy was performed and a panel of 25 tissues collected into 10% neutral buffered formalin for histopathology. Twenty of these tissues were collected without fixation for virological assessment.

### Plasma biochemistry and blood counts

Plasma biochemistry was evaluated with the VetScan Piccolo Xpress® blood analyzer (Abaxis, USA) using a thawed lithium heparin plasma sample (stored at − 80 °C after processing on the day of collection). Evaluation of complete blood counts was performed on the day of sample collection using a VetScan HM5 hematology system (Abaxis, USA) using whole blood collected in EDTA tubes. All analyses were conducted according to the manufacturer’s instructions and values reported in standard international (SI) units.

### Quantification of SARS-CoV-2 viral loads by qRT-PCR

Viral RNA was extracted from nasal washes, oral and rectal swabs, blood and tissues using the Mag-Max Viral RNA isolation kit (Thermo Fisher Scientific). Tissues were weighed, before homogenization in 1 mL of transport media (PBS + 0.1% BSA) using a FastPrep-24 (MP Biomedicals), of which 50 μL was transferred to 260 μL of MagMax buffer for viral RNA extraction. The RNA extraction was then completed on a KingFisher Flex instrument as per the manufacturer’s instructions. The presence of SARS-CoV-2 RNA in blood, oral and rectal swabs, nasal washes, and tissues were evaluated by quantitative reverse transcription PCR (qRT-PCR) targeting the SARS-CoV-2 E gene using an AgPath-ID One-Step RT-PCR kit (Thermo Fisher Scientific) with the following primers and Taqman probe: CoV-E-fwd (5′-AGTACGAACTTATGTACTCATTCGTT-3′), CoV-E-R2 (5′-ATATTGCAGCAGTACG-CACACA-3′) and CoV E Probe 5′-6-FAM-ACACTAGCCATCCTTACTGCGCTTCG-MGB-3′). The cycling conditions were as follows: 45 °C for 10 min, 95 °C for 10 min, followed by 45 cycles of 95 °C for 15 s and 60 °C for 45 s. Positive results were defined by a cycle threshold (Ct) value of ≤ 41. To quantify viral RNA copies, the Ct values from samples were calculated from a standard curve generated from a synthetic standard diluted from 10^2^ to 10^10^ copies per reaction (the estimated limit of detection was 10^4^ copies/mL).

### Quantification of viral loads by TCID_50_ and serology

Samples (plasma, oral and rectal swabs, nasal washes, and tissues) positive by qRT-PCR were tested for live virus by TCID_50_ assay in a 96-well plate format. VeroE6 cells were infected with tenfold serial dilutions of samples in DMEM with 2% FCS and incubated at 37 °C with 5% CO_2_. At four days post-infection, plates were assessed for CPE for the lowest dilution at which 50% of the wells exhibited cytopathic effect. The TCID_50_ was calculated according to the Reed-Muench method^37^. Serum samples collected pre- and post-virus challenge were gamma-irradiated (50 kGy), after which they were heat-inactivated and virus neutralising assays performed as previously described^33^. The neutralisation capacity of serum samples was tested against 100 TCID_50_ of SARS-CoV-2 with the serum/virus mixtures plated on VeroE6 cells and incubated for 3 days at 37 °C with 5% CO_2_ before being scored for CPE. Neutralisation titres were calculated as the highest dilution of serum resulting in a lack of viral CPE.

### Histology and immunohistochemistry

Tissues were fixed in 10% neutral buffered formalin, embedded in paraffin wax using standard procedures, sectioned at 4 µm, and stained with hematoxylin and eosin (H&E) for histopathologic examination. For immunohistochemistry (IHC), dewaxed tissue sections were quenched for 10 min in aqueous 10% hydrogen peroxide. Antigen retrieval was performed by using the Agilent PT Link (Dako) module for 30 min at 97 °C in pH 9 antigen retrieval solution (DM828, Dako). A rabbit polyclonal antibody targeting the nucleocapsid protein (40588-T62, Sino Biological Inc) was used at a dilution of 1:8000 (60 min incubation) to detect SARS-CoV-2 antigen in tissues. Sections were visualised using an Envision Flex horseradish peroxidase (HRP)-secondary antibody (DM822, Dako) for 20 min (goat anti-rabbit and anti-mouse immunoglobulins), chromogen aminoethyl carbazole (AEC), and counterstained with Lillie-Mayer’s Haematoxylin and Scotts Tap Water. Sections were digitised using a Pannoramic Scan II (3DHISTECH Ltd) whole slide imager before photomicrographs were taken using the image capture function of the CaseViewer software.

### Data analysis

Figures were generated using GraphPad Prism 8.4.2 software for Windows (GraphPad Software, San Diego, California USA, www.graphpad.com). Differences between means were evaluated using the paired, two-tailed, Student’s t-test, one-way analysis of variance (ANOVA) or mixed-effects analysis (restricted maximum likelihood) for repeated measures data, with multiple comparison testing where appropriate and were deemed significant at p < 0.05.

## Results

### Study design and clinical findings

**Figure 1:**
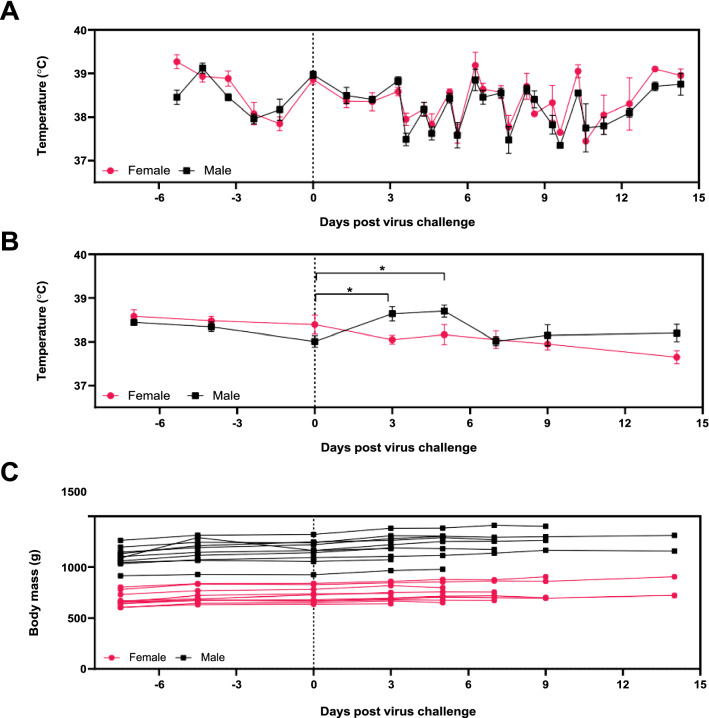
Clinical Observations of ferrets after SARS-CoV-2 challenge. (**A**) Ferrets were observed at least once a day, with twice a day monitoring (approximately 6 h apart) from day 3 onwards. Temperatures were taken during routine clinical monitoring using the identifier microchip on all days apart from the day of virus challenge. Data points indicate mean temperatures ± SEM. (**B**) Rectal temperatures were taken during the enrolment/acclimatisation phase, and at day 3, 5, 7, 9, and 14 while ferrets were anaesthetised. Data points indicate mean rectal temperatures ± SEM. Statistical significance indicated by * (p < 0.05). (**C**) Ferret body weight was recorded while anaesthetised at day − 7, − 4, 0, 3, 5, 7, 9, and 14 days post virus challenge. Points and lines represent individual animals. In each of the panels, black squares indicate male animals and rose-coloured circles indicate female animals.

Ten male and ten female ferrets were randomised into five different groups to determine their serial sacrifice time point (3, 5, 7, 9, or 14 days post-inoculation [p.i.]). All ferrets were inoculated with SARS-CoV-2 (BetaCoV/Australia/VIC01/2020) at a dose of 4.64 × 10 TCID_50_ in a volume of 0.5 mL via the intranasal route. After virus inoculation, ferrets in all groups were monitored for clinical signs of disease. There were no perturbations in clinical score parameters (data not shown) or bodyweight after virus challenge (Fig. 1). Fluctuations in microchip temperatures between morning and afternoon readings were observed (Fig. 1A), with the rectal temperatures providing a better indication of core body temperature (Fig. 1B). For male ferrets, there was a statistically significant elevation in rectal temperature between day of challenge (baseline) vs. day 3 and 5 (p = 0.0234 and 0.0217 respectively) using Tukey’s multiple comparisons test (mixed-effects model). There were no statistically significant differences observed between male and female microchip temperatures following virus challenge, however, at day 3 the average rectal temperature of male ferrets was statistically higher than that in female ferrets (p = 0.0256) [Sidak’s multiple comparisons test]. Body weights of ferrets remained stable after SARS-CoV-2 infection and all ferrets gained weight while on study (Fig. 1C). No ferrets in this work succumbed to infection following infection with SARS-CoV-2 and ferrets were humanely killed at their specified time point.

### Haematology and biochemistry

Four days prior to virus challenge, baseline blood samples were collected for hematology and plasma biochemistry. Hematological analysis revealed an increase in monocytes at day 5 post-inoculation (p.i.) for male and female ferrets compared to pre-challenge values (p = 0.0492 and 0.0163, respectively [unpaired t test] Fig. S1Q). The percentage of monocytes also appeared to be elevated at day 5, however, this was not statistically significant. The two female ferrets humanely killed at day 9 p.i. had a statistically significant reduction in white blood cell numbers, granulocytes and platelet distribution width size (Fig. S1I,M,S, respectively) when compared to the pre-challenge baseline values for female ferrets using one-way ANOVA. Other hematological parameters such as platelets, haemoglobin and lymphocytes were similar to those seen at baseline with no significant gender-specific differences observed either at baseline or post SARS-CoV-2 challenge (Fig. S1).

Changes in several plasma biochemistry markers are shown in Fig. S2. Glucose (GLU) levels following virus infection were generally higher than pre-challenge values (Fig. S2A). These differences were statistically significant in male ferrets on day 7, 9 and 14 post-challenge compared to pre-challenge values (p = 0.00686, 0.00073 and 0.01970 respectively) and at day 9 in female ferrets (p = 0.002921) by a one-way ANOVA with Tukey’s multiple comparison test. Blood urea nitrogen (BUN) levels were generally lower after virus challenge compared to that at baseline, with a statistically significant decrease observed in male ferrets 7 days after virus challenge (p = 0.01897) (Fig. S2B). Creatinine (CRE) levels were also lower compared to the mean values collected pre-challenge and were statistically significant at day 3 for female ferrets (p = 0.01976). Albumin (ALB) plasma concentrations were higher at day 9 and 14 compared to pre-challenge levels (p = 0.01227 and 0.00323 respectively). Aspartate aminotransferase (AST) levels were elevated at day 3 post-infection for both male and female ferrets compared to their pre-challenge values (p = 0.08873 [n.s] and 0.01493 respectively), however, at later time points the levels of AST returned to baseline levels (Fig. S2H). Alkaline phosphatase (ALP) was generally decreased following infection with SARS-CoV-2 between days 3 and 14 compared to pre-challenge values and was statistically lower than baseline values at day 7 for females and day 14 for males (p = 0.01143 and 0.02753 respectively) (Fig. S2I). For other biochemistry markers, there were no significant changes observed and were comparable to baseline levels. An additional comparison of clinical chemistry values for each analyte was performed between male and female ferrets at each study day and no gender-based differences were observed (one-way ANOVA with Tukey’s multiple comparison test). Due to the limited numbers of ferrets and the inherent biological variation between outbred ferrets, the biological significance of these results is currently unknown.

### Virus shedding and presence in internal organs

**Figure 2:**
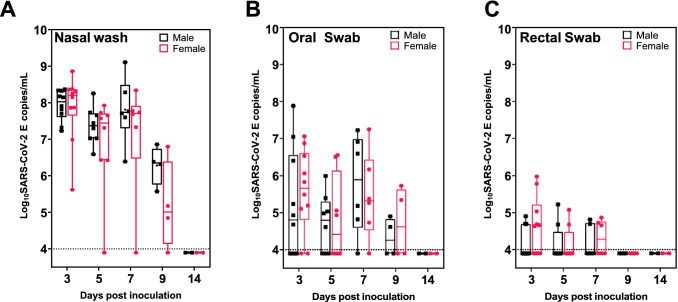
Shedding of SARS-CoV-2 in ferret secretions following intranasal exposure. Virus excretion from (**A**) nasal wash, (**B**) oral swab, and (**C**) rectal swabs at 3, 5, 7, 9, and 14 days post-administration of SARS-CoV-2 as detected by qRT-PCR. Individual data points are plotted, together with box plots show- ing the median values with the whiskers indicating the maximum and minimum viral genome copies/mL. Male data is shown in black and female data in rose. The horizontal dotted line in each panel represents the lower limit of detection.

The presence of SARS-CoV-2 in tissues and organs, and shedding in rectal, oral swabs and nasal washes were evaluated by qRT-PCR and quantification of infectious virus [TCID_50_] in samples that were positive by qRT-PCR (Tables 1 and 2). There was no virus detected in the plasma of ferrets following intranasal challenge of virus. Viral RNA, however, was detected in nasal washes, oral and rectal swabs as of day 3 p.i. (Fig. 2). Copies of SARS-CoV-2 viral genomes decreased in nasal wash specimens at day 5, but were elevated at day 7 p.i., with similar levels of shedding between male and female ferrets (Fig. 2A). Nasal shedding decreased between days 7 and 9, and by day 14 the viral RNA in the nasal wash from the four remaining ferrets (two male and two female) were below detectable levels. Shedding in oral swabs displayed a similar trend to that observed in nasal washes, with median copies/mL values approximately 2–3 logs lower than the nasal wash samples (Fig. 2B). The least amount of viral shedding was observed in rectal swabs with only three out of ten male ferrets and six out of ten female ferrets with detectable viral genomes at day 3 (Fig. 2C). Female ferrets showed higher levels of rectal shedding compared to male ferrets at day 3, however, this was not statistically significant. Shedding was detected up until day 7 in rectal swabs, and at 9 and 14 days p.i., viral genomes were undetectable for all ferrets.

**Table 1:** Detection of SARS-CoV-2 RNA in nasal washes, swabs (oral and rectal), blood and tissues in ferrets infected with SARS-CoV-2 following intranasal challenge.

	Days post inoculation									
	3		5		7		9		14	
Sex	M	F	M	F	M	F	M	F	M	F
No	n = 10	n = 10	n = 8	n = 8	n = 6	n = 6	n = 4	n = 4	n = 2	n = 2
Nasal wash	10/10	10/10	8/8	7/8	6/6	5/6	4/4	3/4	0/2	0/2
Swab oral	6/10	8/10	5/8	4/8	5/6	5/6	2/4	2/4	0/2	0/2
Swab recta	3/10	6/10	2/8	2/8	2/6	3/6	0/4	0/4	0/2	0/2

No	n = 2	n = 2	n = 2	n = 2	n = 2	n = 2	n = 2	n = 2	n = 2	n = 2
Plasma EDT	0/2	0/2	0/2	0/2	0/2	0/2	0/2	0/2	0/2	0/2
Turbinate	2/2	2/2	2/2	2/2	2/2	2/2	1/2	0/2	0/2	0/2
Pharynx	2/2	2/2	2/2	2/2	2/2	2/2	2/2	1/2	0/2	0/2
Trachea	1/2	2/2	1/2	2/2	1/2	0/2	0/2	0/2	0/2	0/2
Lung	0/2	1/2	1/2	0/2	0/2	0/2	0/2	0/2	0/2	0/2
Lymph RETR	2/2	1/2	1/2	1/2	1/2	1/2	1/2	0/2	2/2	1/2
Lymph BRON	0/2	0/2	1/2	0/2	0/1	0/2	0/2	0/2	0/2	0/2
Thymus	0/2	0/2	0/2	0/2	0/2	0/2	0/2	0/2	0/2	0/2
Heart	0/2	0/2	0/2	0/2	0/2	0/2	1/2	0/2	0/2	0/2
Olfac lobe	1/2	2/2	1/2	1/2	1/2	0/2	0/2	0/2	0/2	0/2
Occip lobe	2/2	2/2	1/2	2/2	1/2	0/2	0/2	0/2	0/2	0/2
Stomach	2/2	1/2	2/2	2/2	2/2	2/2	0/2	0/2	0/2	0/2
Duodenum	1/2	1/2	2/2	1/2	1/2	1/2	0/2	0/2	0/2	0/2
Jejunum	0/2	0/2	0/2	0/2	1/2	0/2	0/2	0/2	0/2	0/2
Ileum	1/2	0/2	0/2	0/2	1/2	1/2	0/2	0/2	0/2	0/2
Colon	1/2	0/2	1/2	1/2	2/2	1/2	0/2	0/2	0/2	0/2
Liver	0/2	0/2	0/2	0/2	1/2	0/2	0/2	0/2	0/2	0/2
Kidney	0/2	0/2	0/2	0/2	1/2	1/2	0/2	0/2	0/2	0/2
Spleen	0/2	0/2	0/2	0/2	1/2	0/2	0/2	0/2	0/2	0/2
Ovary	×	0/2	×	0/2	×	0/2	×	0/2	×	0/2
Testicle	0/2	×	0/2	×	1/2	×	0/2	×	0/2	×

For each sample type the numerator shows the number of qRT-PCR positive samples and the denominator shows the total number of samples tested.

*LYMPH RETR* the retropharyngeal lymph node, *LYMPH BRON* the bronchial lymph node, *OLFAC LOBE* the olfactory bulb, *OCCIP LOBE* the occipital lobe respectively. × indicates not applicable.

**Table 2:** Recovery of infectious virus from qRT-PCR positive shedding samples and tissues after in- tranasal challenge of ferrets with SARS-CoV-2.

	Days post inoculation									
	3		5		7		9		14	
Sex	M	F	M	F	M	F	M	F	M	F
No	n = 10	n = 10	n = 8	n = 8	n = 6	n = 6	n = 4	n = 4	n = 2	n = 3
Nasal wash	4/10	6/10	4/8	2/7	3/6	1/5	0/4	1/3	–	–
Swab oral	1/6	1/8	0/5	0/4	1/5	0/5	0/2	0/2	–	–
Swab recta	0/3	0/6	0/2	0/2	0/2	0/3	–	–	–	–

No	n = 2	n = 2	n = 2	n = 2	n = 2	n = 2	n = 2	n = 2	n = 2	n = 2
Plasma EDT	–	–	–	–	–	–	–	–	–	–
Turbinate	2/2	1/2	2/2	2/2	2/2	2/2	0/1	–	–	–
Pharynx	1/2	0/2	0/2	1/2	0/2	1/2	0/2	0/1	–	–
Trachea	0/1	0/2	0/1	0/2	0/1	–	–	–	–	–
Lung	–	0/1	0/1	–	–	–	–	–	–	–
LYMPH RETR	0/2	0/1	0/1	0/1	0/1	0/1	0/1	–	0/2	0/1
LYMPH BRON	–	–	0/1	–	–	–	–	–	–	–
Thymus	–	–	–	–	–	–	–	–	–	–
Heart	–	–	–	–	–	–	0/1	–	–	–
OLFAC LOBE	0/1	0/2	0/1	1/1	0/1	–	–	–	–	–
OCCIP LOBE	0/2	0/2	0/1	0/2	0/1	–	–	–	–	–
Stomach	0/2	0/1	0/2	0/2	0/2	0/2	–	–	–	–
Duodenum	0/1	0/1	0/2	0/1	0/1	0/1	–	–	–	–
Jejunum	–	–	–	–	0/1	–	–	–	–	–
Ileum	0/1	–	–	–	0/1	0/1	–	–	–	–
Colon	0/1	–	0/1	0/1	0/2	0/1	–	–	–	–
Liver	–	–	–	–	0/1	–	–	–	–	–
Kidney	–	–	–	–	0/1	0/1	–	–	–	–
Spleen	–	–	–	–	0/1	–	–	–	–	–
Ovary	×	–	×	–	×	–	×	–	×	–
Testicle	–	×	–	×	0/1	×	–	×	–	×

The numerator indicates the number of samples that were positive by infectivity assay (> 92.8 TCID_50_/mL). The denominator indicates the number of qRT-PCR positive samples tested. × indicates not applicable.

*LYMPH RETR* the retropharyngeal lymph node, *LYMPH BRON* the bronchial lymph node, *OLFAC LOBE* the olfactory bulb, *OCCIP LOBE* the occipital lobe respectively.

Infectious virus was recovered from qRT-PCR positive nasal wash samples (21/53) (Table 2) and was detected as soon as day 3 p.i. at 928 TCID_50_/mL for one male and one female ferret (data not shown). The remaining infectious nasal wash samples were below the limit of quantitation (BLOQ, estimated to be greater than 92.8 but less than 632 TCID_50_/mL), and detected between 3 and 7 days post-challenge. The one exception was a female ferret that had an infectious nasal wash sample at day 9 p.i. (Table 2). Only three of the 37 qRT-PCR positive oral swabs yielded infectious virus and these were from two male ferrets, one at day 3 and another male ferret at day 7, and one female ferret at day 3 (BLOQ) (Table 2). No infectious virus was recovered from the 18 qRT-PCR positive rectal swabs (Table 2).

**Figure 3:**
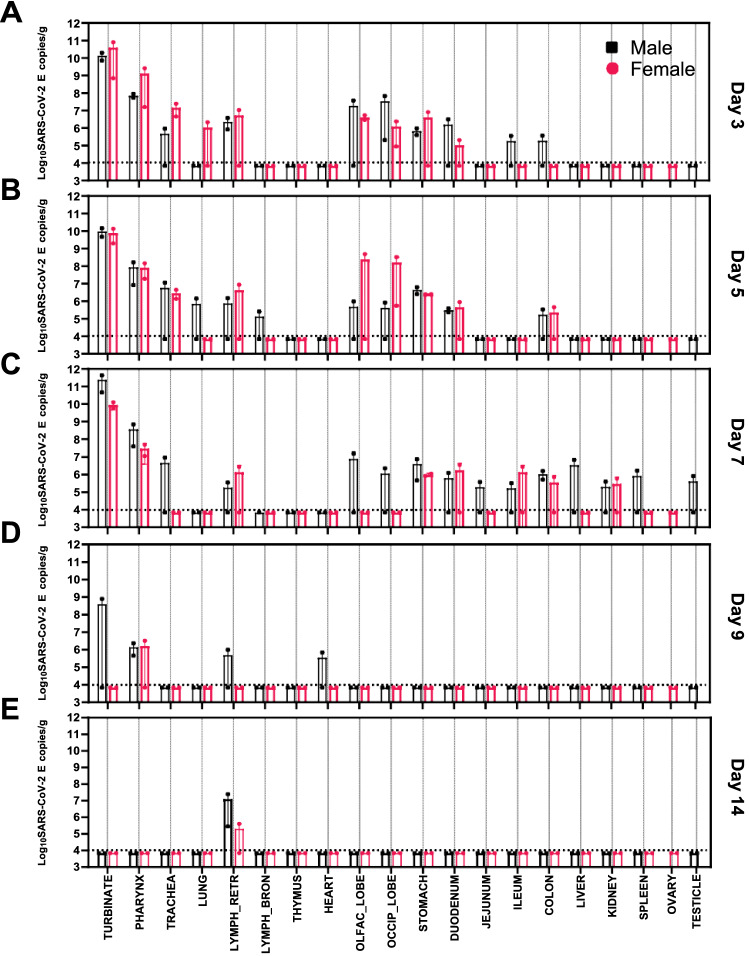
Distribution of SARS-CoV-2 RNA in ferret tissues and organs after intranasal challenge. Log_10_ SARS-CoV-2 viral genomes per gram of tissue detected in organs or tissues of ferrets inoculated with SARS-CoV-2 Australia/VIC01 isolate. Panel (**A**) shows data from two male and two female ferrets euthanased on day 3, (**B**) day 5, (**C**) Day 7, (**D**) day 9, and (**E**) day 14 respectively. Bars indicate mean values, together with individual data points shown as black squares for males and rose-coloured circles for female ferrets, error bars indicate ± SEM. The horizontal dashed line shows the lower limit of detection. LYMPH RETR represents the retropharyngeal lymph node, LYMPH BRON the bronchial lymph node, OLFAC LOBE the olfactory bulb, and OCCIP LOBE the occipital lobe respectively.

Of all the tissues and organs tested, nasal turbinates had the highest levels of SARS-CoV-2 as detected by qRT-PCR and infectivity assays (Fig. 3). At day 3, 5, and 7 p.i., on average approximately 10^10^ copies/g of tissue was detected in the nasal turbinates of ferrets, with only one male ferret still positive for SARS-CoV-2 by qRT-PCR at day 9 in the turbinate sample (Fig. 3A–D). Infectious virus was recovered from the turbinate tissues (Table 2), with the highest titers recovered at day 7, with 1.22 × 10^6^ TCID_50_/g of tissue from one female ferret and 8.85 × 10^5^ TCID_50_/g from a male ferret (data not shown). Apart from these two turbinate samples, infectious virus was recovered from nine other qRT-PCR positive nasal turbinates and were between 4.2 × 10^3^ and 5.11 × 10^4^ TCID_50_/g of tissue (across days 3–7 p.i.). In the respiratory tract, the next most virus abundant tissues were the pharynx and retropharyngeal lymph nodes, followed by the trachea and lung (Fig. 3). Of the qRT-PCR positive respiratory tissues, low levels of infectivity (BLOQ) were recovered from only three of 15 pharynx samples, one at days 3 (male), 5 (female), and 7 (female) respectively (Table 2). Interestingly, SARS-CoV-2 viral RNA persisted in the retropharyngeal lymph nodes up to 14 days p.i. even though all other tissues and organs were negative for viral RNA (Fig. 3). No infectious virus was recovered from the viral RNA-positive retropharyngeal lymph nodes, trachea or lung samples between days 3 and 14.

When the olfactory bulb and occipital lobe of the brains were examined for SARS-CoV-2 viral RNA, both male and female ferrets showed detectable viral genomes at day 3 and 5 p.i. (Fig. 3). At day 3, three out of four ferrets (two female and one male) were positive for viral genomes in both the olfactory bulb and occipital lobe, with the remaining male ferret showing viral RNA positivity in the occipital lobe only and not the olfactory bulb. At day 5, both female ferrets had viral genomes detected in the occipital lobe, but only one with olfactory bulb positivity. On day 5 and day 7 only a single male out of each pair at sacrifice was positive by qRT-PCR, and in both cases, the olfactory bulb and occipital lobe were positive (˜ 10^6^ copies/g of tissue). Viral RNA was not detected in the brain of female ferrets from day 7 to 14. Infectious virus was detected in one olfactory bulb sample collected at day 5, however no infectious virus was recovered from any of the qRT-PCR positive occipital lobe samples (Table 2).

SARS-CoV-2 viral RNA was detected in the stomach and other parts of the gastrointestinal (GI) tract, such as the duodenum, jejunum, ileum, and colon. Male and female ferrets all had similar levels of recoverable viral RNA (approximately 3 × 10^6^ copies/g) from stomach tissue at days 3, 5, and 7, apart from one female ferret which was undetectable on day 3 (Fig. 3A–C). Viral RNA was detected in the duodenum of ferrets at days 3, 5, and 7 in both male and female ferrets, but in most cases just one out of each pair, except for day 5 where both male ferrets were positive (Table 1). The distribution of virus to the jejunum and ileum was less consistent, with only one jejunum sample from a male ferret at day 7 being positive out of the twenty animals that were screened by qRT-PCR across the 14 days (Table 1). Three ileum samples were positive for SARS-CoV-2 RNA and two of these were in ferrets sacrificed at day 7 (one male and one female), the remaining sample from a day 3 ferret. The colon tissue collected on day 7 had slightly higher levels of viral RNA compared to those from day 3 and 5 but an average of all qRT-PCR positive colon samples yielded 6.35 × 10^5^ copies/g. One of the male ferrets sacrificed at day 7 had detectable virus in the liver, kidney, spleen, and testis (Fig. 3C). Only one other female ferret had detectable virus in the kidney and this was also on day 7 (Fig. 3C). No infectious virus was recovered from any of the GI tract tissues, kidney, spleen, or testis (Table 2).

Serological testing confirmed that all pre-challenge test sera lacked SARS-CoV-2 neutralising activity. All other terminal serum samples collected after viral challenge were negative (< 1:10) for SARS-CoV-2 neutralisation, except for one female ferret at 14 days post-inoculation which had a neutralisation titre of 1:10 (data not shown).

### Histopathology and immunohistochemistry

**Figure 4:**
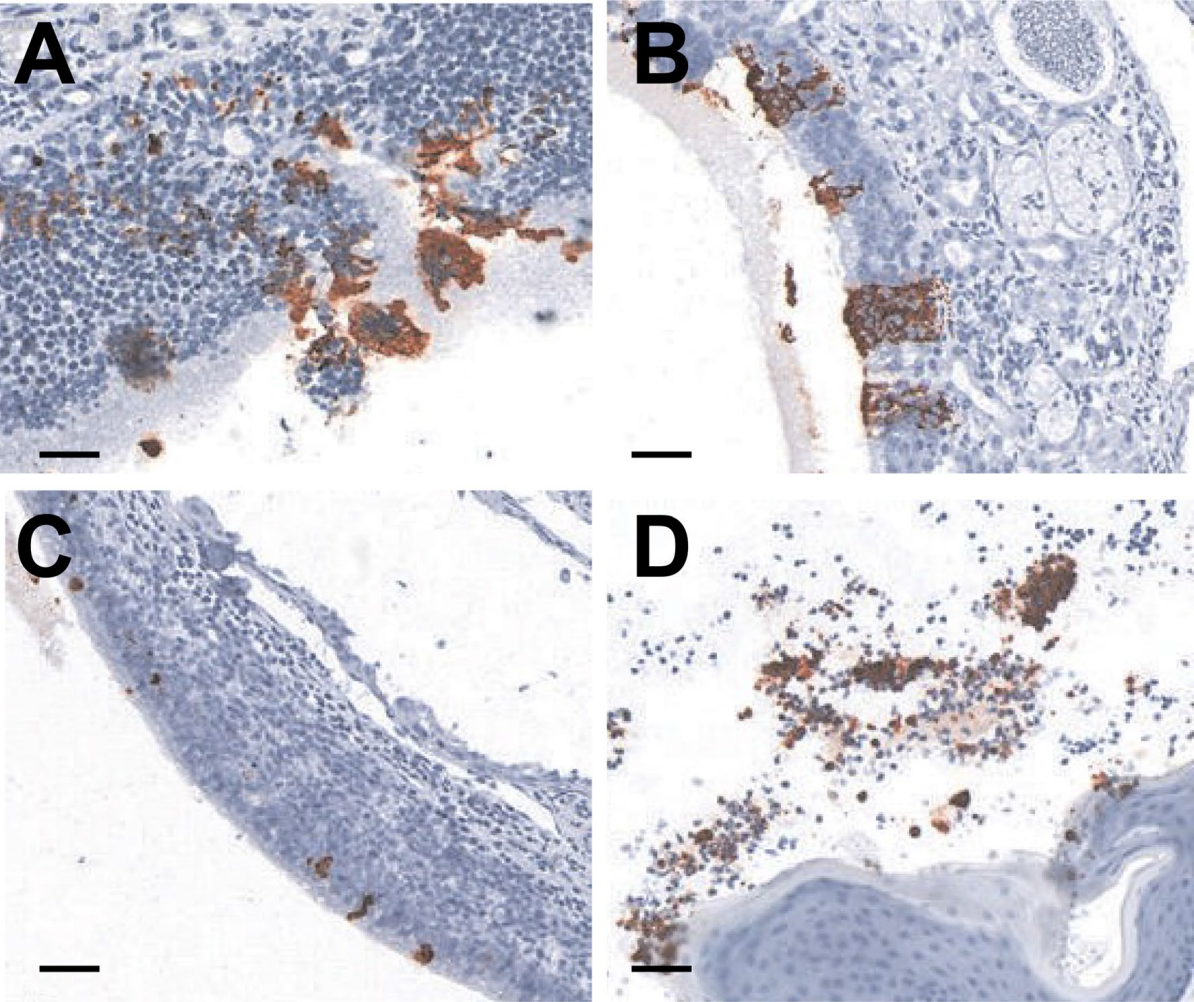
SARS-CoV-2 nucleocapsid IHC labeling in nasal turbinates. (**A**) Representative image from a ferret euthanased at day 3 post-inoculation (p.i.), intense positive cytoplasmic diffuse labeling in olfactory epithelium. (**B**) Day 5 p.i., small patches of positively labeled olfactory epithelium. (**C**) Day 7 p.i., scattered individual antigen-positive epithelial cells in the respiratory epithelium of the nasal turbinates. (**D**) Day 7 p.i., antigen-positive sloughed epithelial cells admixed with many inflammatory cells and mucus overlying the oropharyngeal mucosa. SARS-CoV-2 IHC sections are labelled with a nucleocapsid- specific polyclonal antibody, HRP-secondary antibody and aminoethyl carbazole chromagen. Scale bars in each panel represent 50 µm.

**Figure 5:**
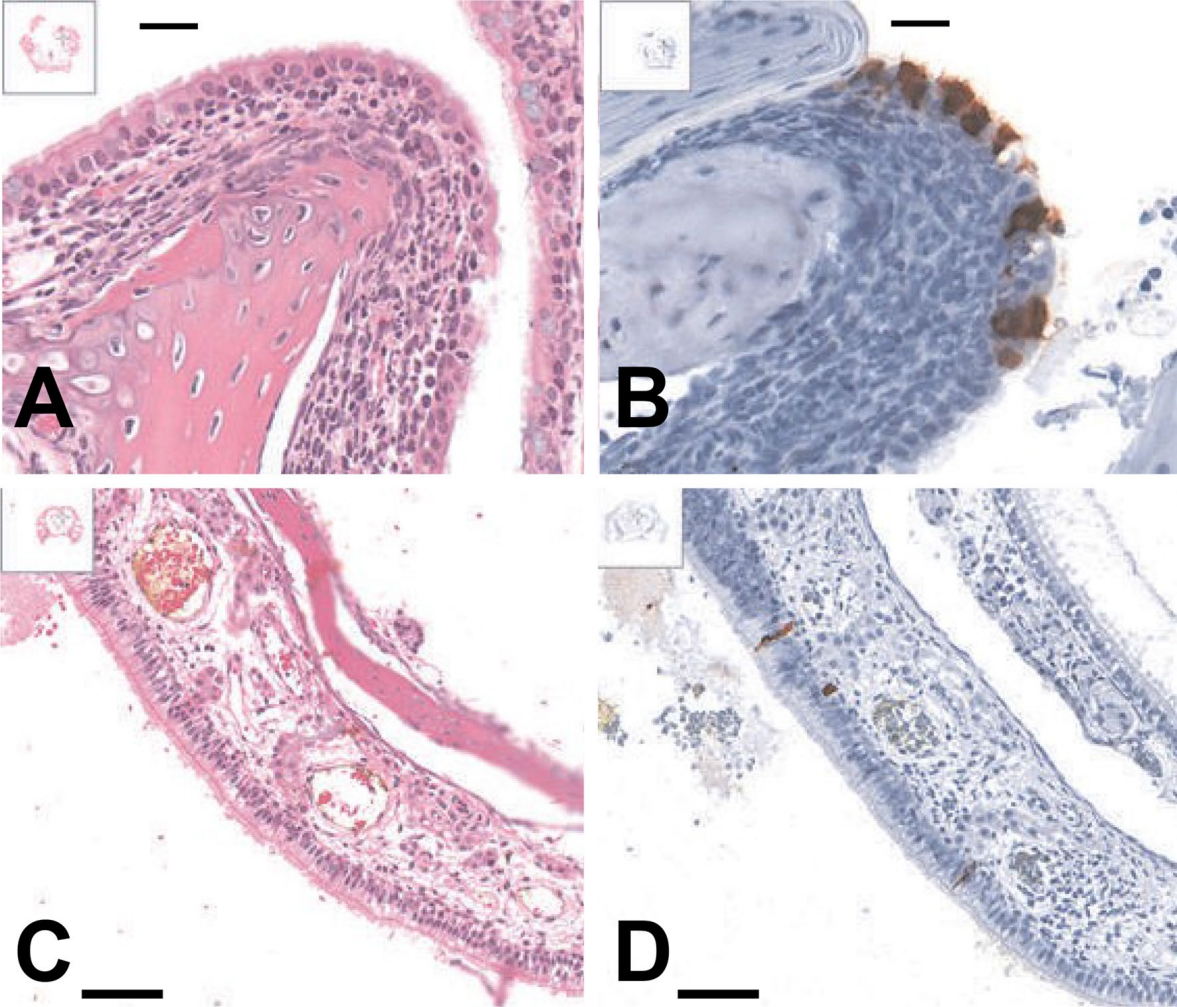
Positive SARS-CoV-2 antigen labeling was not associated with a specific inflammatory response in the nasal respiratory epithelium. (**A**, **B**) show nasal turbinates from a ferret euthanased at day 3 post-inoculation (p.i.) with moderate infiltration of neutrophils and lymphocytes in an antigen-positive region of the respiratory epithelium. Tissues from a ferret humanely killed at 7 days p.i., (**C**, **D**) show an antigen-positive region of the respiratory epithelium, not associated with an inflammatory infiltrate. Panels (**A**, **C**) represent H&E stained sections. Panels (**B**, **D**) represent the corresponding SARS-CoV-2 IHC sections labelled with a nucleocapsid-specific polyclonal antibody, HRP-secondary antibody and aminoethyl carbazole chromagen. Scale bars in each panel represent 50 µm.

In the current study, the histopathological and SARS-CoV-2 immunohistochemical profiles of SARS-CoV-2 challenged ferrets were assessed across the time points, day 3, 5, 7, 9, and 14 p.i. Consistent with the data on the recovery of infectious virus from nasal turbinates, the SARS-CoV-2 nucleocapsid antigen was detected up to day 7 p.i. and largely restricted to the nasal epithelium (Fig. 4). Antigen load was generally low and viral antigen was sparsely distributed in both the olfactory and respiratory epithelium of the nasal turbinates (Fig. 4B and Table 3). Antigen-positive cells in the olfactory epithelium were typically not associated with a significant inflammatory response, although minimal to mild leucocytic infiltrate was noted in a small subset of ferrets (Fig. 5A,B). Specific inflammatory reaction against infected respiratory epithelial cells could not be assessed with great confidence due to the presence of a non-specific neutrophilic and/or lymphoplasmacytic infiltrate affecting both antigen-positive and antigen-negative areas and antigen-negative animals. Furthermore, antigen-positive areas without associated inflammatory infiltrate were also observed (Fig. 5C,D). However, if present, a specific inflammatory response against SARS-CoV-2 infection in the respiratory epithelium would be expected to be minimal due to the limited extent of the viral infection. Sloughed epithelial cells in oropharyngeal exudate were also positive in one ferret (Fig. 4D), and weakly labelled round cells were observed in the retropharyngeal lymph node of another. SARS-CoV-2 nucleocapsid antigen was not detected in the remaining organs.

**Table 3:** Incidence of SARS-CoV-2 antigen-positive nasal turbinates and antigen scores. NA, no antigen-positive cells detected in any of the ferrets at the respective time points; Antigen scoring system: 1, less than 50 positive cells per section; 2, 50–100 positive cells per section; 3, > 100 positive cells per section.

Day p.i	Number of antigen-positive ferrets (n = 4 per time point)	Antigen score of ferrets (1, 2 or 3)
3	3/4	2, 1, 2
5	4/4	1, 1, 1, 1
7	3/4	1, 1, 2
9	0/4	NA
14	0/4	NA

In a subset of ferrets, varying degrees of neutrophilic, eosinophilic, lymphoplasmacytic and/or histiocytic infiltrate was observed in the trachea and lungs. However, these histologic changes were not associated with corresponding positive SARS-CoV-2 antigen labelling, and these are therefore interpreted to be incidental changes that were independent of SARS-CoV-2 replication.

## Discussion

Here we demonstrate that the VIC01 isolate of SARS-CoV-2 infects the upper respiratory tract of ferrets with consistent viral RNA shedding in nasal wash samples and oral swabs up until day 9 post intranasal challenge. Although clinical signs of infection were not observed in ferrets, infectious virus was recovered in nasal wash samples, oral swabs, nasal turbinates, pharynx samples, and one olfactory bulb sample. By day 14, there was no further viral excretion in this model, indicating that infection was eventually controlled, mimicking cases of mild or asymptomatic infection observed in humans.

At present, several experimental models of SARS-CoV-2 infection have been developed in ferrets suitable for vaccine and therapeutic testing. Using the same VIC01 isolate as described in this paper, a dose- dependent study of SARS-CoV-2 was conducted by Ryan et al., 2021^32^. In that study, consistent viral RNA shedding was detected in the upper respiratory tract of ferrets intranasally challenged with high (5 × 10^6^ pfu) and medium (5 × 10^4^ pfu) doses of VIC01. In both groups, significant lung pathology and post-viral fatigue were observed, findings that were not present in our ferret model. One consideration is that our intranasal challenge dose (4.64 × 10^4^ TCID_50_) was likely to be 1.5 times lower than that of their medium-dose group based on the approximation of 1 pfu = 0.7 × TCID_50_. Even though high titres of virus (up to 1.22 × 10^6^ TCID_50_/g of tissue) were recovered from nasal turbinate tissues in our study 5–7 days post intranasal inoculation, we only recovered low levels of live virus in nasal washes and oral/throat swabs, similar to the model described by Ryan et. al., 2021.

Apart from the upper respiratory tract, we detected viral RNA in the olfactory bulb, and occipital lobe of ferrets between day 3 and 7 p.i. and a range of other tissues of the gastrointestinal tract, as well as kidney, spleen and testes. One male ferret had detectable SARS-CoV-2 in the heart by qRT-PCR at day 9, and three out of four ferrets (2 males and 1 female) had a retropharyngeal lymph node sample that was positive for viral RNA at day 14. This distribution of VIC01 viral RNA was more widespread compared to the ferret model established by Shi et al.^30^, which used isolates F13-E and CTan-H, collected from an environmental sample in the Huanan Seafood Market in Wuhan, and a human patient respectively. Ferrets that were intranasally infected with 10^5^ pfu of F13-E or CTan-H and euthanized at 4 days p.i., had detectable viral RNA present in the nasal turbinates, soft palate and tonsils, but not in the trachea, lung, heart, liver, spleen, kidneys, pancreas, small intestine or brain. Overall, the VIC01 isolate was detectable by qRT-PCR in a wider range of tissues, including the brain of ferrets as early as day 3 p.i., in contrast to ferrets administered with higher doses of F13-E and CTan-H.

In our study, VIC01 was recovered from nasal turbinates, pharynx samples and one olfactory lobe tissue sample, however, we failed to recover infectious virus from trachea or lung samples that were positive by qRT-PCR. A ferret-to-ferret transmission model by Kim et al.^28^, using strain NMC-nCoV02 (isolated from a South Korean patient with COVID-19) established the presence of viral RNA in nasal turbinate, trachea, lung, kidney and intestine samples at 4 and 8 days post intranasal inoculation with 3 × 10^5^ TCID_50_ of virus. Infectious virus was recovered only from nasal turbinates at day 4 and 8 (3/3 ferrets), in lung samples at day 4 p.i. (2/3 ferrets), and in 1/3 trachea samples at day 8 p.i. By day 12, there was no viral RNA detected in these tissues and generally, these findings were consistent with the detection of VIC01 viral genomes in our model between day 3 and 7 p.i., one notable difference being that we did not recover infectious virus from the trachea or lung tissues for the VIC01 isolate.

With respect to viral detection of SARS-CoV-2 in the blood of infected ferrets, we examined plasma from ferrets between day 3 and 14 and were unable to detect VIC01 viral genomes by qRT-PCR. Kim et al., detected low levels of SARS-CoV-2 spike RNA in sera of inoculated ferrets at day 2 and 4 p.i. with 0.35 log_10_ copies/mL of viral RNA recovered at both time points. Richard et al., who used the VIC01 isolate in their ferret model, also failed to detect virus in the blood despite using higher doses of challenge inoculum. Taken together, viremia does not appear to be a significant feature of SARS-CoV-2 infection in ferrets and reflects the low percentage of viremia observed in human patients with COVID-19^38–40^.

Serological testing of the terminal serum samples collected after SARS-CoV-2 challenge revealed that only one of the four ferrets at day 14 had low levels of neutralising antibodies (1:10). Serum collected at day 3, 5, 7 and 9 days post-challenge were all negative (< 1:10). This finding was in agreement with studies by Ryan et al., and Everett et al., which used the VIC01 isolate of SARS-CoV-2 and where neutralising antibody levels were generally low. Everett et al., detected virus-specific antibodies against the spike protein and nucleocapsid protein of SARS-Cov-2 in ferrets 21 days post-infection, with a low neutralizing antibody response detected in two of four ferrets^34^.

Even though COVID-19 patients typically present with signs of respiratory disease, several case studies have reported gastrointestinal symptoms and/or evidence that some patients have viral RNA or live infectious virus in the feces^41^. The fecal–oral route of transmission of SARS-CoV-2 is yet to be confirmed^42^; however, in this study, viral RNA was distributed throughout the GI tract, involving the stomach, duodenum, jejunum, ileum and colon in both male and female ferrets between day 3 and 7 p.i. after intranasal administration of the VIC01 strain. Given that ferrets that had qRT-PCR positive rectal swabs also had positive nasal wash samples and/or oral swabs, the detection of viral RNA in the GI tract may be due to the swallowing of viral particles from the upper respiratory tract. Other possibilities may be the detection of remnants of infected submucosal intestinal antigen-presenting immune cells or that there is active virus replication in intestinal cells, although viral antigen was not detected in the intestine by IHC. This suggests that if active viral replication was present in the intestine, it was minimal below the level of detection by IHC and virus isolation.

The blood biochemistry and hematology results observed in this ferret model aligned with some changes observed in COVID-19 patients, for example, increases in aspartate aminotransferase (AST) and glucose were observed in 35% and 52% of patients admitted with SARS-CoV-2 pneumonia^43^. However, ferrets in this study exhibited only a mild clinical course, and the mechanism behind these parameter changes may not necessarily mimic that in the human disease course. Furthermore, the inherent variation in the level of stress experienced by the different ferrets, and the use of sedatives, such as xylazine and ketamine, known to produce hyperglycemia^44,45^, may offer alternative explanations to the observed changes in serum glucose. The mechanism behind the AST elevation is also undetermined, but individual variation (including sample collection variation causing hemolysis) and/or background subclinical hepatic pathology should be considered as possible explanations for this change. Chen et al., found that the absolute value of lymphocytes in most patients was reduced, and this was consistent in this study with decreased numbers observed 3–14 days p.i. compared to baseline levels (Fig. S1O). One limitation of this study was that blood samples were not collected longitudinally from the same animals at each time point, only pre- and post-infection, with the latter collected as a terminal sample.

The detection of virus in the olfactory bulb and occipital lobe of ferrets in this model highlights the potential for neuronal involvement following SARS-CoV-2 infection. Despite the recovery of live virus in one ferret from the olfactory bulb and viral RNA in the occipital lobe as early as 3–7 days post-infection, no neurological signs and neuropathology were observed during this study. Numerous reports of the neurological manifestations of COVID-19 are emerging with some patients showing non-specific symptoms such as headache and confusion, and others with specific signs such as seizure or cerebrovascular problems^46^. Human coronavirus OC43 has been implicated in a case of fatal encephalitis in an 11-month old boy with severe combined immunodeficiency^47^. The mechanism by which SARS-CoV-2 enters the brain, and whether this virus can lead to encephalitis in immunocompromised individuals will require further research. SARS-CoV-2 challenge in experimentally immunosuppressed ferrets may present one avenue to address this question. The predilection of viral replication for the nasal epithelium, particularly the olfactory epithelium, suggests that our model may be appropriate to study the mechanism of anosmia associated with SARS-CoV-2 infection in humans as well as the process of subclinical neuroinvasion.

Here we describe the pattern of viral shedding in a time-course study of SARS-CoV-2 infection in ferrets. Viral excretion data and immunohistochemistry findings suggest that the current ferret model system is appropriate for studying the mechanism of subclinical SARS-CoV-2 infections and assessing the efficacy of vaccines or therapeutic agents on viral load. The ferret model provides a straightforward way to measure the efficacy of vaccines through the reduction in virus excretion, while ferret sera can also be used to screen for efficacy against new variants of SARS-CoV-2. Given the importance of the nasal turbinates as the predominant site of infection in ferrets, they may also be a good model to assess intranasal vaccines^48^. Immunological studies are underway to better understand the host response to SARS-CoV-2 infection in ferrets and provide the foundation for vaccine efficacy studies.

## Supplementary Information

Supplementary Information.
