# Supplementary material for: Characterisation and natural progression of SARS-CoV-2 infection in ferrets
Source: Sci Rep. 2022 Apr 5;12:5680. doi: 10.1038/s41598-022-08431-6 (PMC8981194; doi:10.1038/s41598-022-08431-6)
Supplement: Supplementary file 1 — Supplementary Information. [file 41598_2022_8431_MOESM1_ESM.pdf]

Supplementary Material

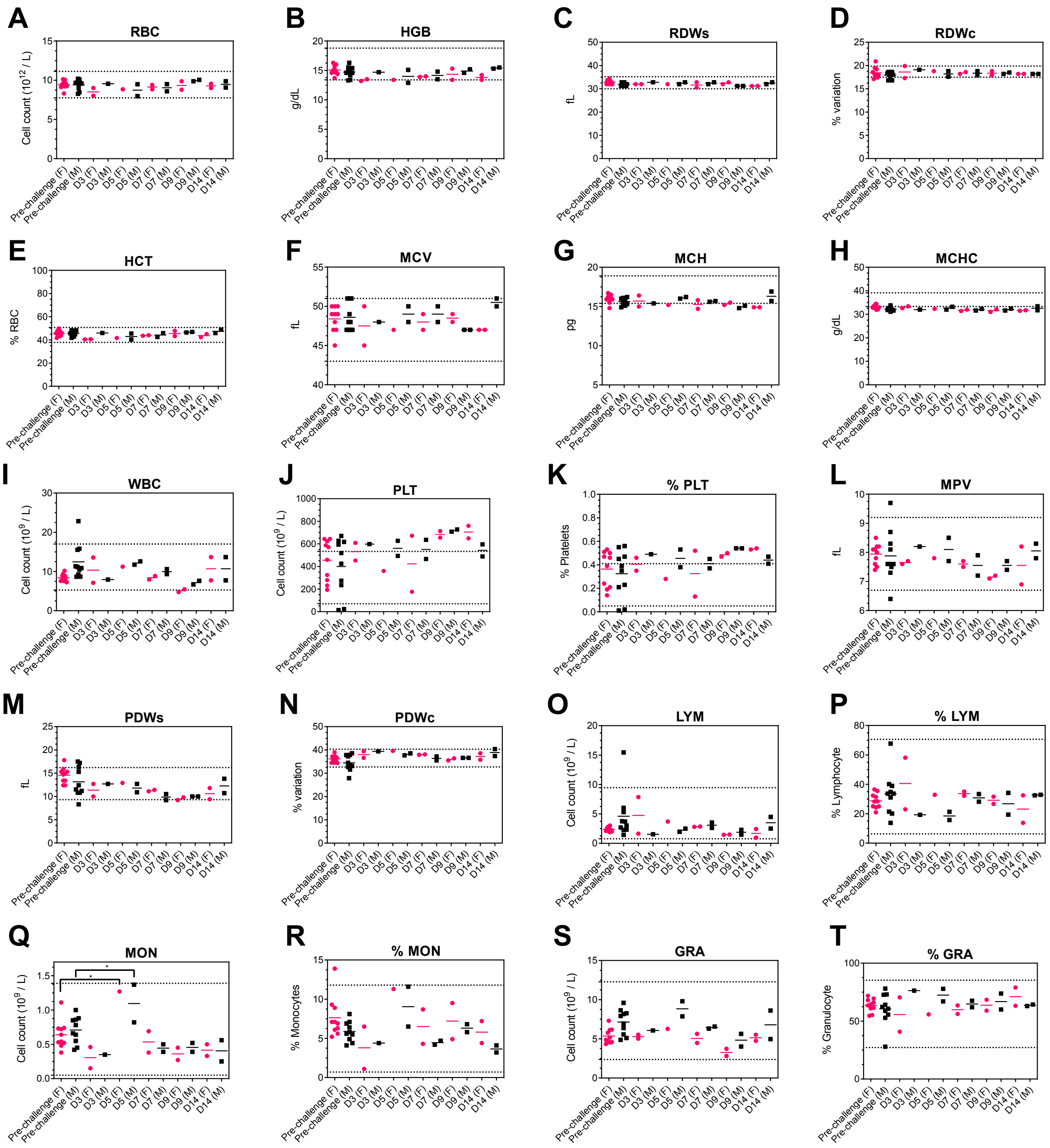

Figure S1: Hematological parameters of SARS-CoV-2 infected ferrets. Each of the panels are as follows, (**A**) red blood cell count (RBC), (**B**) hemoglobin (HGB), (**C**) red blood cell distribution width - size (RDWs), (**D**) red blood cell distribution width - variation (RDWc), (**E**) hematocrit (HCT), (**F**) mean corpuscular volume (MCV), (**G**) mean corpuscular hemoglobin (MCH), (**H**) mean corpuscular hemoglobin concentration (MCHC), (**I**) white blood cell count (WBC), (**J**) platelet count (PLT), (**K**) plateletcrit (%PLT) (**L**) mean platelet volume (MPV), (**M**) platelet distribution width - size (PDWs), (**N**) platelet distribution width - variation (PDWc), (**O**) lymphocytes (LYM), (**P**) % lymphocytes of total cells (% LYM), (**Q**) monocytes (MON), (**R**) % monocytes of total cells (%MON), (**S**) granulocytes (GRA), (**T**) % granulocytes (%GRA). Black squares and rose-coloured circles represent individual values of male and female samples, bars represent mean for each group, dotted lines represent upper and lower reference ranges. Stasticical significance indicated by * where $p < 0.05$.

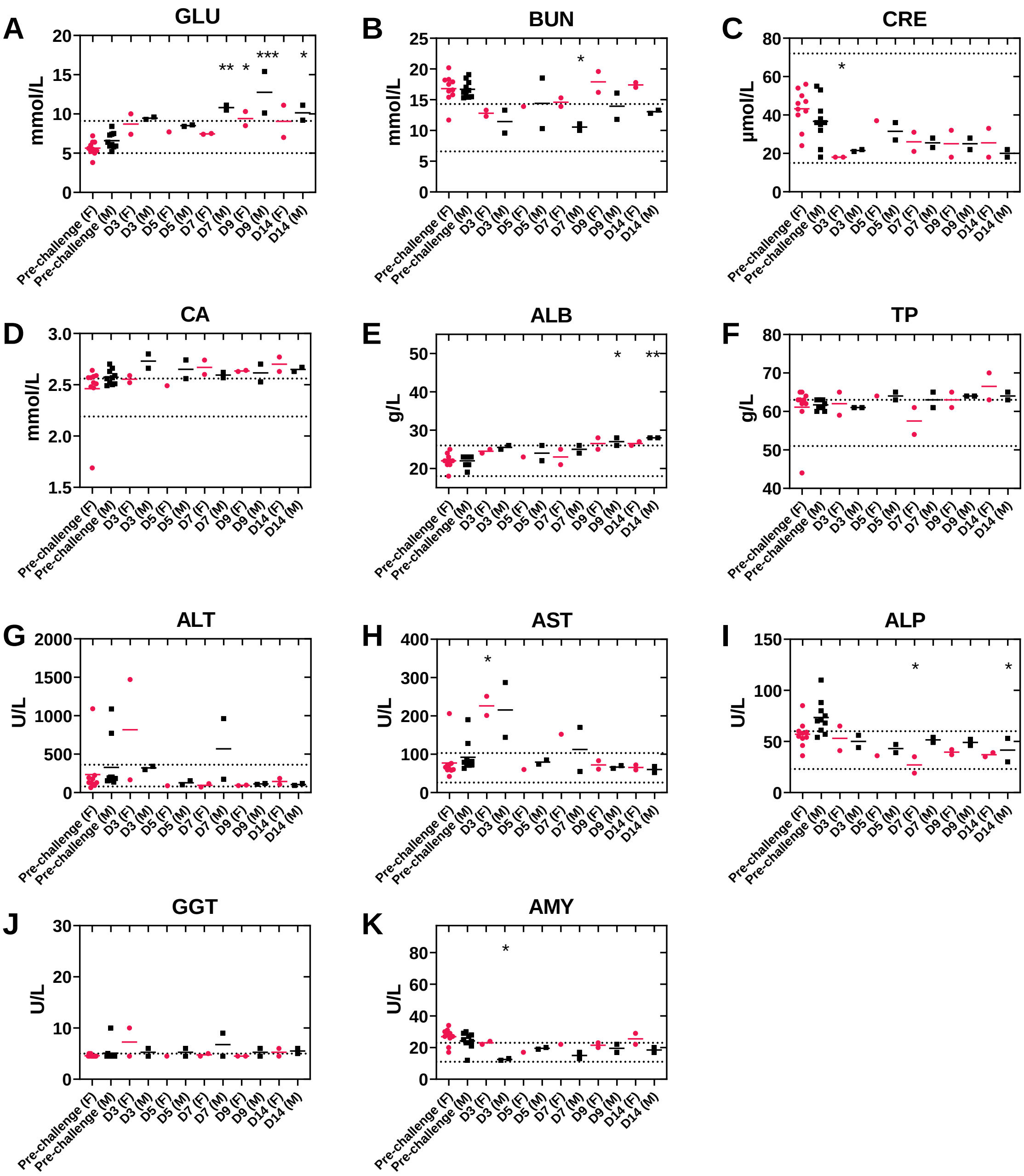

Figure S2: Blood biochemistry in SARS-CoV-2-infected ferrets. Individual biochemistry values in male and female ferrets pre- and post-challenge with SARS-CoV-2 for (**A**) glucose (GLU), (**B**) blood urea nitrogen (BUN), (**C**) creatinine (CRE), (**D**) calcium (CA), (**E**) albumin (ALB), (**F**) total protein (TP), (**G**) alanine aminotransferase (ALT), (**H**) aspartate aminotransferase (AST), (**I**) alkaline phosphatase (ALP), (**J**) gamma glutamyltransferase (GGT), and (**K**) amylase (AMY). Black squares and rose-coloured circles represent individual values of male and female samples, bars represent mean for each group, dotted lines represent upper and lower reference ranges, dashed line for GGT represents lower limit of detection. Statistical significant indicated by * $0.01 < p < 0.05$, ** $0.001 < p < 0.01$, *** $p < 0.001$.
